# The Plastic Deformation Mechanism in Nano-Polycrystalline Al/Mg Layered Composites: A Molecular Dynamics Study

**DOI:** 10.3390/nano14010114

**Published:** 2024-01-02

**Authors:** Zhou Li, Tong Shen, Xiao Hu, Lu Zhang, Xianshi Jia, Jiaqing Li, Che Zhang

**Affiliations:** 1 College of Mechanical and Electrical Engineering, Central South University, Changsha 410083, China; lizhou_industry@hotmail.com (Z.L.); 213711051@csu.edu.cn (T.S.); yjyhuxiao@pzhsteel.com.cn (X.H.); zhangl@csu.edu.cn (L.Z.); 221026@csu.edu.cn (X.J.); 2 State Key Laboratory of Precision Manufacturing for Extreme Service Performance, Changsha 410083, China; 3 College of Chemical Engineering, Fuzhou University, Fuzhou 350116, China; 4 Department of Mechanical Engineering, University of Melbourne, Parkville, VIC 3010, Australia

**Keywords:** Al/Mg layered composites, plastic deformation mechanism, molecular dynamics simulation, intermetallic compounds

## Abstract

Understanding plastic deformation behaviour is key to optimising the mechanical properties of nano-polycrystalline layered composites. This study employs the molecular dynamics (MD) simulation to comprehensively investigate the effects of various factors, such as grain sizes, strain rates, and the interlayer thicknesses of the intermetallic compounds (IMCs), on the plastic deformation behaviour of nano-polycrystalline Al/Mg layered composites. Our findings reveal that the influence of grain size on deformation behaviour is governed by the strain rate, and an increase in grain size is inversely proportional to yield stress at low strain rates, whereas it is positively proportional to tensile stress at high strain rates. Moreover, an optimal thickness of the intermediate layer contributes to enhanced composite strength, whereas an excessive thickness leads to reduced tensile strength due to the fewer grain boundaries (GBs) available for accommodating dislocations. The reinforcing impact of the intermediate IMCs layer diminishes at excessive strain rates, as the grains struggle to accommodate substantial large strains within a limited timeframe encountered at high strain rates. The insights into grain sizes, strain rates, and interlayer thicknesses obtained from this study enable the tailored development of nanocomposites with optimal mechanical characteristics.

## 1. Introduction

Layered metal composites offer several advantages by preserving and combining the positive attributes of single metals or alloys, resulting in a superior comprehensive performance [1,2,3]. The integration of different metallic materials [4,5,6] and preparation processes [7,8,9,10] can effectively enhance their strength, toughness, stiffness, impact resistance, wear resistance, and electrical conductivity [11,12,13,14,15,16]. Employing nano-polycrystalline layered composites offers a substantial enhancement of the material’s performance, and this improvement stems from the remarkable strength exhibited by nano-scale grains, surpassing that of coarse crystalline materials with equivalent compositions by several magnitudes [17,18]. Among these nano-polycrystalline layered composites, Al/Mg composites have gained widespread use in the automotive and aerospace sectors, particularly in light weighting for aerospace conveying equipment and automotive components [19,20,21].

The knowledge of plastic deformation mechanisms assists in designing and developing new nano-polycrystalline multi-layered composites with improved properties [22,23]. By understanding how different factors, such as layer thickness, composition, and interface characteristics, influence the plastic deformation, strategies can be developed to enhance the composite’s resistance to deformation, and the microstructure and properties of composites can be tailored to meet specific application requirements [24,25]. Extensive research efforts have been devoted to investigating the plastic deformation behaviour of materials on the nanoscale under various conditions, including grain size [26,27,28,29,30,31,32], strain rate [31,32,33], grain boundaries (GBs), and interfacial interactions [5,23,34,35,36,37,38]. For instance, Liu [30] utilised the molecular dynamics (MDs) method to examine the plastic deformation phenomenon of nano-polycrystalline Mg, and observed the inverse Hall–Petch relation at the grain sizes below 10 nm. Xue et al. [38] investigated the deformation process in nano-polycrystalline Mg through MD simulations and transmission electron microscope (TEM) experiments. They observed the transformation of a hexagonal close-packed (HCP) structure into a face-centred cubic (FCC) structure during the compression process, with the activation of GBs-assisted deformation as the grain size decreased. Bian et al. [34] studied the effect of interfaces on the plastic deformation of the polycrystalline Cu/Al systems, and observed the gradient distribution of shear strain at the Al_2_Cu interface, which enhanced the material’s strength and toughness. Zhang et al. [23] examined the deformation interaction mechanism at the Ti/Al HCP-FCC interface, reporting the generation of twinning between FCC grains caused by the emission of Shockley dislocations and full dislocations at the P-type HCP/FCC interface. Li et al. [36,37] found, through a series of studies, that the presence of H at the GBs of polycrystalline Fe and Ni and the behaviour of bias aggregation both significantly affect the dislocation mechanism and are accompanied by GB embrittlement and decohesion. Other studies investigated the deformation behaviour of various metallic materials, such as Ti/TiN nanolaminates [39], Cu/Rb laminates [40], and Cu/Ag multilayers [41], using MD simulations.

Most of the previous studies have primarily focused on examining single nano-polycrystalline metal materials and the impact of a few variables on the mechanical properties of nano-composites. However, it is crucial to recognize that material-related deformation at the nanoscale is a complex process influenced largely by multiple variables. To obtain a comprehensive understanding of the deformation behaviour, it is necessary to investigate the combined effects of these key variables simultaneously, and this study directs its attention to the remarkable characteristics of nano-polycrystalline Al/Mg layered composites. By utilising the MDs method, we explore the concurrent influence of grain size, interlayer thickness of intermetallic compounds (IMCs), and strain rate on the tensile plastic deformation behaviour of these composites. The research findings not only provide valuable theoretical insights into the study of Al/Mg composites but also address the research gap created by the challenges associated with experimental investigations. This study aims to shed light on the intricate nature of material deformation at the nanoscale and its practical implications in the development of high-performance multi-layered nanocomposites.

## 2. Materials and Methods

The pure polycrystalline Al, Mg, and polycrystalline layered Al/Mg composites were modelled using the open-source Atomsk package [42]. To ensure grain homogeneity, the Voronoi tessellation method [43] was used to randomly generate grains. The deformation behaviour of the polycrystalline system was simulated using the open-source Lammps software (LAMMPS 64-bit, version 3Nov2022-MPI; Simulation tool for particle-based materials modeling at the atomic, meso, and continuum scales; Sandia National Laboratories: Los Alamos, NM, USA, 2022) [44], and the results were analysed and visualised with Ovito [45,46], using the dislocation analysis (DXA) method and the common neighbour analysis (CNA) method. In Liu [30] and other recent studies, the Hall–Petch effect has been observed in Al and Mg polycrystalline models with a grainsize under 10 nm during tensile loading. The simulation boxes for polycrystalline Al and polycrystalline Mg were constructed with 10 nm length in each direction, containing 2, 4, 8, 16, and 32 grains at random, respectively. The dimensions of layered Al/Mg polycrystals were 20 nm × 10 nm × 20 nm, with 4, 8, 16, 32 and 64 grains included; the corresponding grain sizes are 7.94 nm, 6.29 nm, 5 nm, 3.97 nm and 3.15 nm, respectively. The IMCs interlayer is amorphous, and given that the Al/Mg heterogeneous interface is dominated by Al_12_Mg_17_, different thicknesses of IMCs interlayers with a ratio of Al to Mg of 12:17 were mixed using the Lammps script, and an Al/Mg polycrystalline model with an interlayer was generated by using Atomsk to stack Al, IMCs interlayers, and Mg models. The IMCs of Al_12_Mg_17_ were set with thicknesses of 1.25 nm, 2.5 nm, and 3.75 nm for the interlayers, and the polycrystals were generated randomly using the Voronoi method without a specific orientation. Since the modulation period has a significant impact on the mechanical properties of the nano-multilayer film [47,48], the total thickness of the layered Al/Mg composite model will not be changed. 

**Figure 1:**
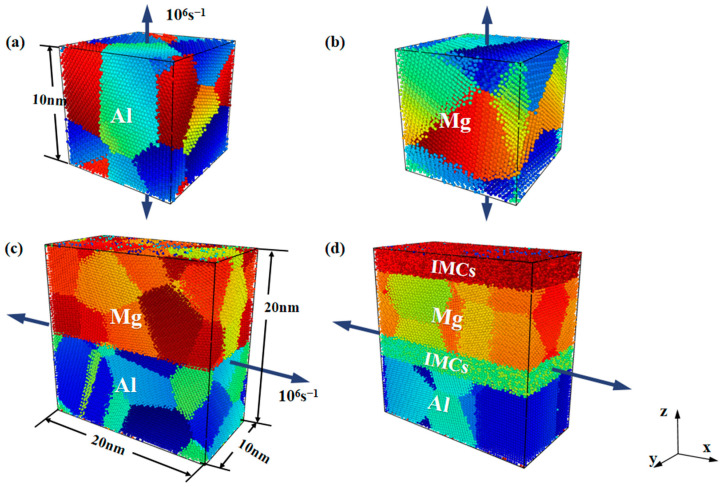
Simulation models and the loading method for (**a**) polycrystalline Al, (**b**) polycrystalline Mg, (**c**) Al/Mg layered composites with no IMCs interlayer, and (**d**) Al/Mg layered composites with IMCs interlayer.

For MDs simulations of metallic polycrystalline systems, the modified embedded atom method (MEAM) electron potential has been commonly used. Although the embedded atom method (EAM) electron potential has a faster simulation speed, it is not an accurate description of the stacking behaviour of Mg polycrystalline [49,50]. Earlier studies have also confirmed that the MEAM electron potential is superior to the EAM electron potential when describing crystal lattice changes and the elastic behaviour of crystals [51]. As such, a MEAM electron potential developed by Kim et al. [52] was adopted in the present study, which has been proven to be effective in describing the deformation behaviour of Al/Mg polycrystalline systems [53]. Periodic boundary conditions were imposed in all three directions, and energy minimisation was performed using the conjugate gradient (CG) algorithm. The system’s temperature was controlled at 300 K in NVT ensemble using the Nose–Hoover method, and the Mg and Al polycrystalline models were loaded at a strain rate of 10^6^ s^−1^ in the vertical direction, and the Al/Mg layered composite models with/without IMCs interlayers were loaded at the strain rates of 10^6^ s^−1^, 10^7^ s^−1^ and 10^8^ s^−1^, respectively, along the parallel heterogeneous interface, as shown in Figure 1.

## 3. Results and Discussion

### 3.1. Mechanical Response and Structural Evolution of Al/Mg Composite with Varying Grain Sizes

**Figure 2:**
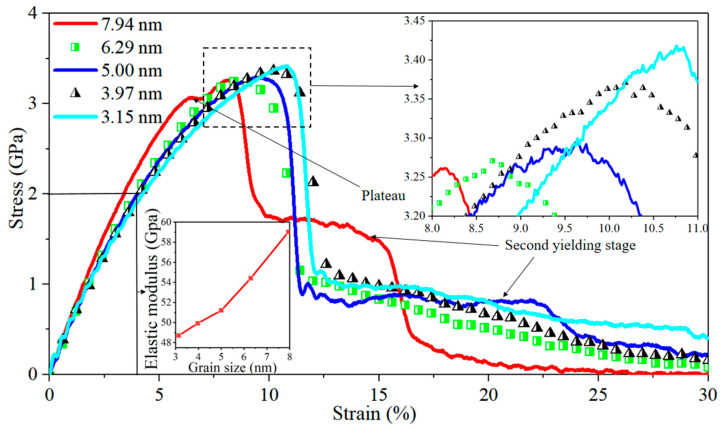
Stress–strain curves for nano-polycrystalline Al/Mg layered composites with different grain sizes when subjected to tensile loading parallel to the interface.

Stress–strain curves for Al/Mg layered composites with varying grain sizes of 3.15 nm, 3.97 nm, 5 nm, 6.29 nm, and 7.94 nm at the strain rate of 10^6^ s^−1^ are demonstrated in Figure 2. The trend indicates that, prior to reaching the yielding point, the elastic modulus of the composites increases with an increase in grain size, whereas the toughness and yield strength of these composites decrease as the grain size increases. Within the composite featuring a grain size of 7.94 nm, the stress–strain curve reaches a plateau at a strain of 6.4%, and the stress reaches its peak before sharply declining at a strain of 8%. Subsequently, at a strain of 10%, the stress plateaus again, marking the onset of the second yield stage. The second yield stage also appears at a strain of 12.3% in the composite with a grain size of 5 nm. The plateau in the stress–strain curve at the strain of 6.4% is thought to be caused by large stacking faults that form within the grain with a grain size of 7.94 nm, and the stress trend does not immediately change under the effect of a stronger Al side.

**Figure 3:**
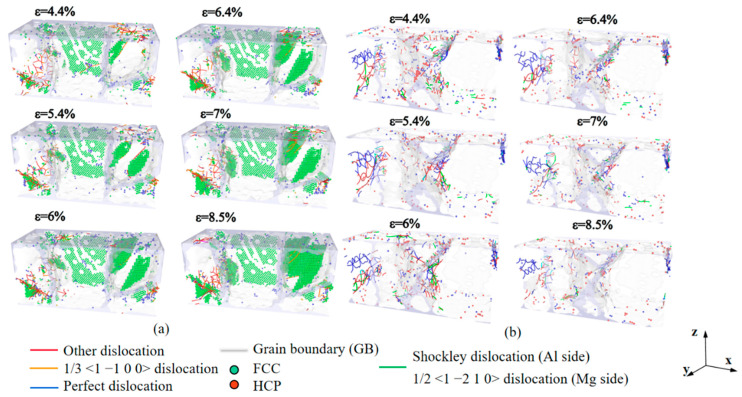
Stacking faults and dislocations in Al/Mg layered composites with a grain size of 7.94 nm at the sides of (**a**) Mg and (**b**) Al, respectively.

Stacking faults and dislocations in Al/Mg layered composites with a grain size of 7.94 nm are shown in Figure 3. A small number of 1/3 <1 −1 0 0> dislocations and other dislocations, which mostly form at the GBs, accompany the stacking faults across the grain. At a strain of 6.4%, the stacking faults do not significantly expand, and the dislocation density at the GBs is higher than at the previous stage, but the dislocation density remains constant at this stage. At a strain of 8.5%, a significant new layer of stacking faults is formed within the large-sized grains on the right side (Figure 3a). There are no obvious stacking faults on the Al side during the tensile loading process, as shown in Figure 3b, and the main dislocations during the deformation process are Shockley dislocations, Perfect dislocations, and a few other dislocations, all of which are distributed at the GBs and do not pass through the grains. The results suggest a correlation between stress fluctuations in the stress rise section and the presence of significant stacking faults on the Mg side, while the composite with smaller grain size has almost no plateau and secondary yielding due to the high proportion of GBs and the low stress concentration. Due to asynchronous fracture, which is essentially non-existent in composites with smaller grain sizes but has little effect on the overall trend of deformation, long GBs of large grain sizes cause a second yielding phase. As this phenomenon of secondary yielding is more pronounced at larger grain sizes, such as 5 nm and 7.94 nm, exploring the structural evolution of a layered composite between relatively large and small grain sizes is essential.

**Figure 4:**
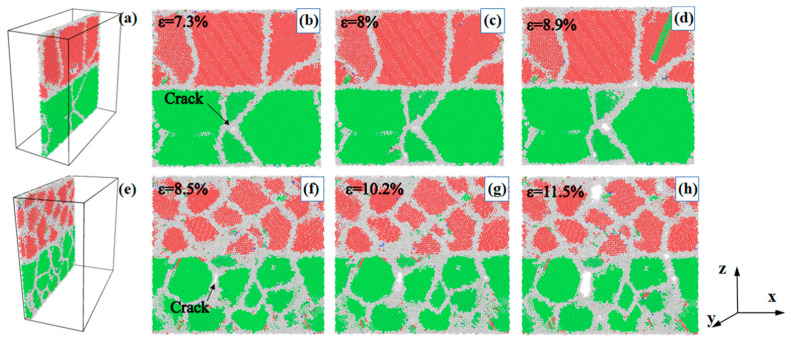
Crack initiation and propagation in nano-polycrystalline Al/Mg layered composites in a (**a**,**e**) the specific region measuring 1 nm in thickness and grain sizes of (**b**–**d**) 7.94 nm and (**f**–**h**) 3.97 nm.

The Al/Mg layered composites with grain sizes of 7.94 nm and 3.97 nm, loaded at the strain rate of 10^6^ s^−1^, are observed to compare the extension of cracks in the composites, as shown in Figure 4. In order to provide a clear demonstration and comparison of the behaviour of the cracks, a specific region of the initial crack area measuring 1 nm in thickness is intercepted (Figure 4a,e) for observation at the moment of initial crack generation, the moment of yield stress, and the moment of steep stress drop corresponding to the strain of the composite, respectively. The composite with a grain size of 7.94 nm exhibits an initial crack at a strain of 7.3% and a yield stress at a strain of 8% without a significant crack expansion, but the crack appears at a strain of 8.9% along with large stacking faults within the grain and a steep drop in stress, as shown in Figure 4b–d. In contrast, the composite with a grain size of 3.97 nm shows an initial crack at 8.5% strain, a yield stress at 10.2% with little change in cracking, and an enlarged crack at 11.5% with a steep drop in stress, as shown in Figure 4f–h, which are all later than in the composite with a grain size of 7.94 nm, with almost no stacking faults within the grain and dislocations distributed at GBs. It is demonstrated that, in Al/Mg layered composites with grain sizes less than 10 nm, the grains characterised by larger sizes significantly influence the coordination of the deformation process. This influence stems from the presence of fewer GBs, resulting in more concentrated stress areas and an uneven stress distribution. Consequently, these composites exhibit reduced strength and toughness, as indicated by previous research [1]. Conversely, the smaller-sized grains predominantly coordinate the deformation through the GBs, which are more abundant in this context. This abundance leads to a more uniform distribution of stress and imparts higher strength and toughness to the composites.

**Figure 5:**
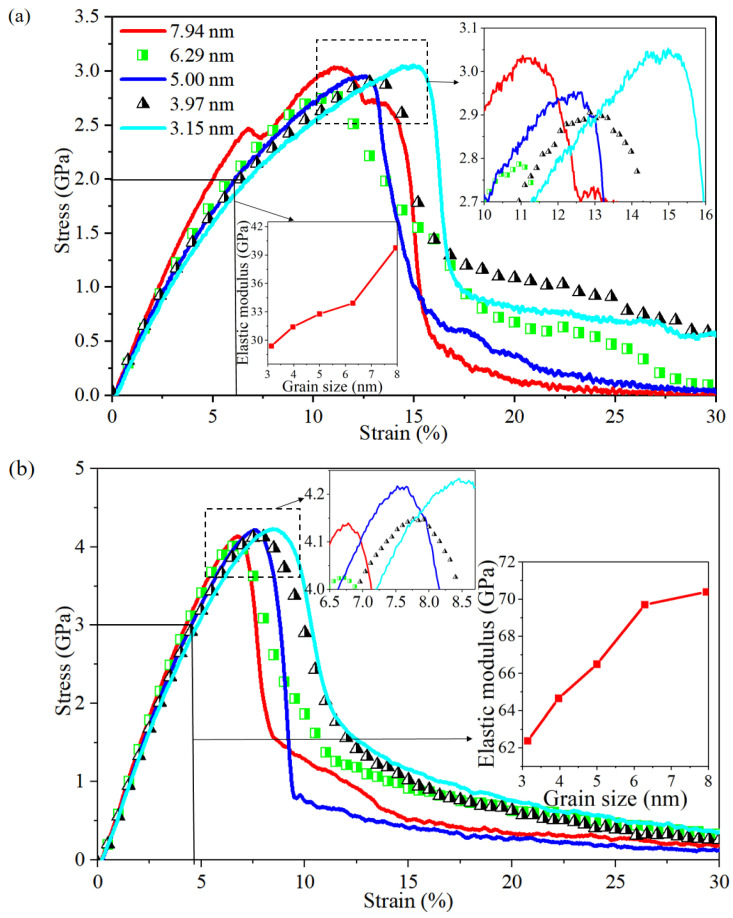
Stress–strain curves with different grain sizes for nano-polycrystalline (**a**) Mg and (**b**) Al, respectively.

**Figure 6:**
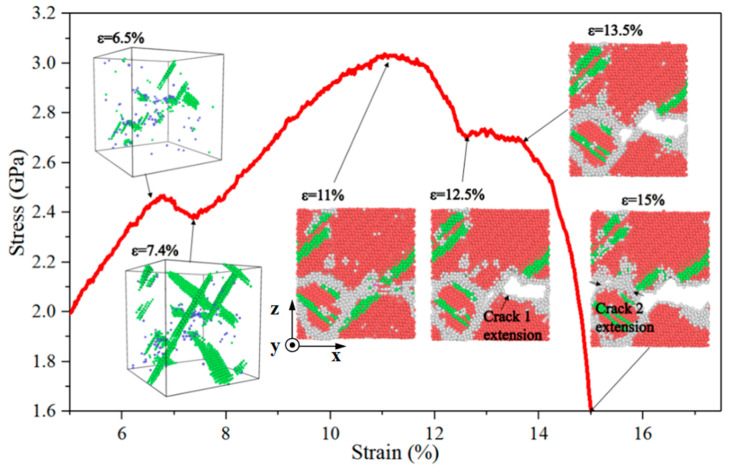
Structural evolution of polycrystalline Mg with a grain size of 7.94 nm in the stress fluctuation section.

Separate polycrystalline Mg and Al with different grain sizes, at a strain rate of 10^6^ s^−1^, were used to examine the potential influence of concurrent tension on the deformation behaviour and structural evolution of bonded Al and Mg, and the resulting stress–strain curves are presented in Figure 5a,b. It can be seen that, contrary to the study by Liu et al. [30], no clear relationship between yield stress and grain size is observed for neither polycrystalline Mg nor Al at relatively low strain rates, while the elastic modulus rises with increasing grain size and the toughness increases with decreasing grain size. Similar to the stress fluctuations in the stress rise section of the Al/Mg layered composites mentioned earlier, the structural evolution of polycrystalline Mg is further analysed, as shown in Figure 6, where the stacking faults within the grains at a strain of 6.5% have been nucleated, the stress first reaches the peak, and then the stress decreases. Contrary to the Mg side of the Al/Mg layered composite, where stacking faults appear prior to the stress fluctuations, the stress decreases when the strain is 7.4% at the bottom and the stacking faults within the grain of polycrystalline Mg immediately expand and grow until they fill the entire grain. At a strain of 11% to 12.5%, the stress starts to drop, and crack 1 (marked by an arrow in Figure 6) develops and extends. At a strain of 12.5% to 13.5%, the stress reaches a plateau, and the crack is largely unexpanded at this stage. The stress drops abruptly at strains above 13.5%, and crack 2 (marked by arrows in Figure 6) has extended by a strain of 15%. It is found that, in Al/Mg layered composites, the fluctuations in the stress-rise section of the large-grain-size composite are influenced by the large stacking faults on the Mg side, with some delay in the effects of Mg due to the presence of Al with a higher elastic modulus on the other side. The stress fluctuations in the stress drop section are related to the stress concentration in the large grain sizes, and the Hall–Petch phenomenon occurs in the composites at low strain rates, whereas it does not occur in polycrystalline Mg and polycrystalline Al.

### 3.2. Influence of the Interlayer on the Plastic Deformation Behaviour

**Figure 7:**
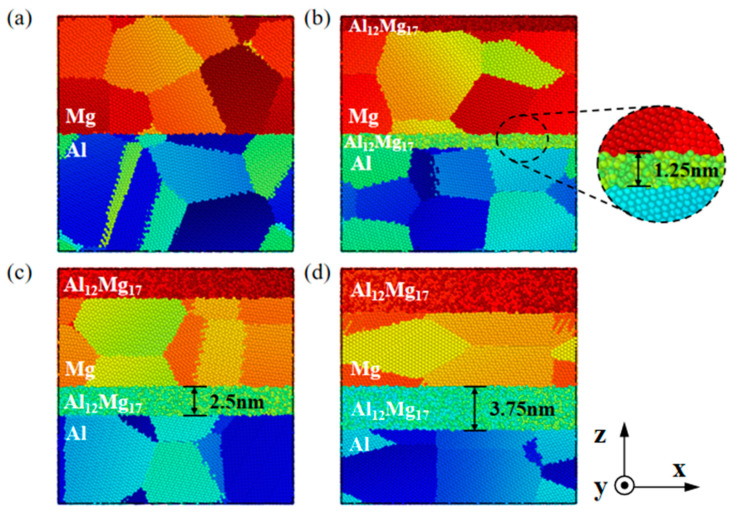
Models of nano-polycrystalline Al/Mg layered composites with various interlayer thicknesses of (**a**) 0 nm, (**b**) 1.25 nm, (**c**) 2.5 nm, and (**d**) 3.75 nm.

The construction of nanoscale polycrystalline Al/Mg layered composites with a well-defined and high-quality heterogeneous interface presents a challenging task due to the interdiffusion of Al and Mg, which typically results in the formation of IMCs. The nanoscale interlayer of IMCs can enhance the shear strength in the parallel interface direction due to the gradient effect [34]. Therefore, it is necessary to investigate the influence of IMCs on the mechanical properties of the nano-polycrystalline layered composites. Nano-polycrystalline Al/Mg layered composites with IMC interlayer thicknesses of 0 nm, 1.25 nm, 2.5 nm, and 3.75 nm were established, and Al_12_Mg_17_ was considered as the main IMC appearing in the preparation of Al/Mg layered composites [54], as shown in Figure 7. This approach allows for a clear demonstration of the effect of IMCs thickness on the deformation behaviour of composites.

**Figure 8:**
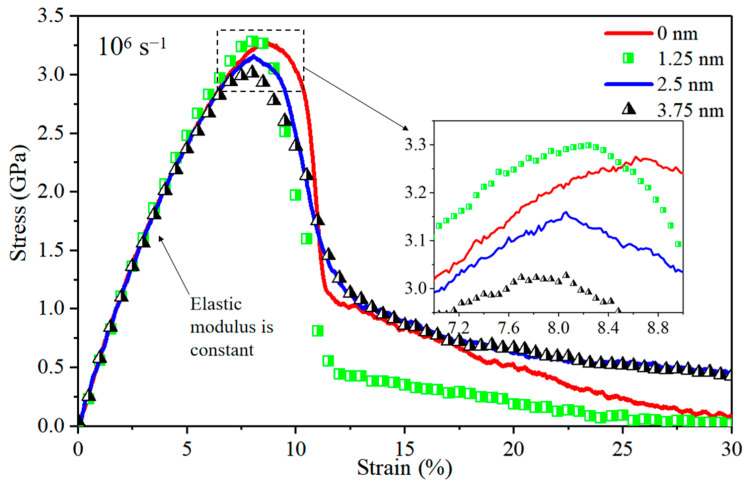
Stress–strain curves for nano-polycrystalline Al/Mg layered composites with various thicknesses in the IMCs interlayer.

**Figure 9:**
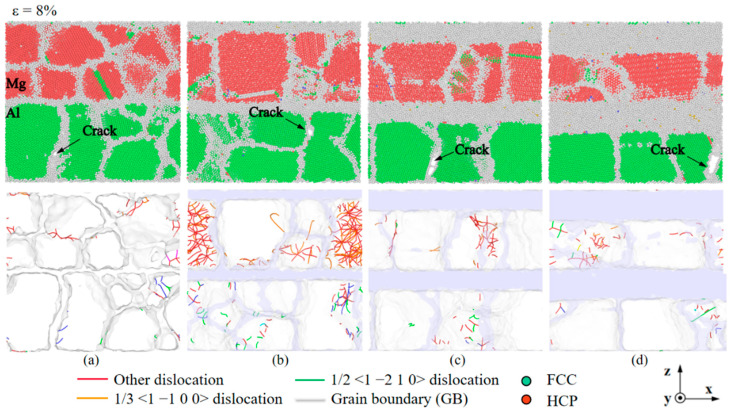
Cracks and dislocations within Al/Mg layered composites at a strain of 8% with IMCs thicknesses of (**a**) 0 nm (**b**) 1.25 nm (**c**) 2.5 nm, and (**d**) 3.75 nm, respectively.

The stress–strain curves for Al/Mg layered composites with IMCs interlayer thicknesses of 0 nm, 1.25 nm, 2.5 nm, and 3.75 nm are shown in Figure 8, respectively. It is noted that the elastic modulus appears to be constant across all composites. In comparison to the composite without an IMCs interlayer, the composite with a 1.25 nm interlayer exhibits a slight increase in yield stress, but the yield stress values exhibit a decreasing trend for the composites with larger interlayer thicknesses (2.5 nm and 3.75 nm). As illustrated in Figure 9, specific crack sections of 1 nm thickness are intercepted from Al/Mg layered composites at a strain of 8% with IMCs interlayer thicknesses of 0 nm, 1.25 nm, 2.5 nm, and 3.75 nm, respectively. It can be seen that the cracks firstly arise at the GBs on the Al side of the vertical heterogeneous interface, and as the thickness of the IMCs interlayer increases, the GBs which can coordinate the deformation become shorter and the crack length increases as a proportion of the GB length. As shown in Figure 9a, the dislocation density at the GBs of the composite without IMCs is low; however, the composite possessing an interlayer thickness of 1.25 nm exhibits a higher capacity for accommodating dislocations within the GBs as depicted in Figure 9b. Conversely, Figure 9c,d illustrate that fewer GBs accommodate fewer dislocations, resulting in a diminished ability to impede crack propagation. Although the IMCs interlayer is able to block dislocations and has a gradient effect in reducing stress concentration [34] at the modulation period of 20 nm, the negative effect of the reduced GBs ratio on the yield stress is greater than the positive effect of the IMCs when the thickness of IMCs exceeds 1.25 nm, resulting in a reduced yield stress.

**Figure 10:**
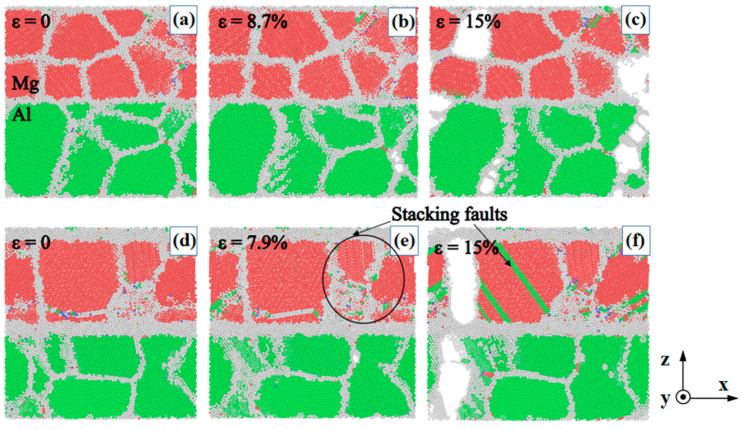
Crack initiation and propagation in nano-polycrystalline Al/Mg layered composites with two IMCs interlayer thickness values of (**a**–**c**) 0 nm, and (**d**–**f**) 1.25 nm.

To investigate the effect of the IMCs interlayer on the deformation behaviour and crack extension tendency of the composites, crack initiation and propagation in nano-polycrystalline Al/Mg layered composites without interlayer and with IMCs interlayer thickness of 1.25 nm are shown in Figure 10. The composite lacking the IMCs interlayer has few stacking faults inside the grains and fractures that shatter over the heterogeneous interface (Figure 10a–c). In contrast, when the composite with a 1.25 nm IMCs interlayer is loaded to peak stress, an increase in stacking faults within the grains is observed (Figure 10d,e). However, this increase is not significant, and after the peak stress, with a further increase in strain, the composite suddenly produced large stacking faults within the grains. At a strain of 15%, both sides of the Al and Mg fracture completely (Figure 10c), but the harder IMCs interlayer does not fracture. This indicates that the gradient effect of the IMCs interlayer has played a role in moderating the stress concentration on both sides of the heterogeneous interface. The grains on both sides of the interface can better coordinate the deformation, and the harder IMCs interlayer can help prevent crack extension.

### 3.3. Strain-Rate-Induced Deformation Mechanisms in Al/Mg Composite

**Figure 11:**
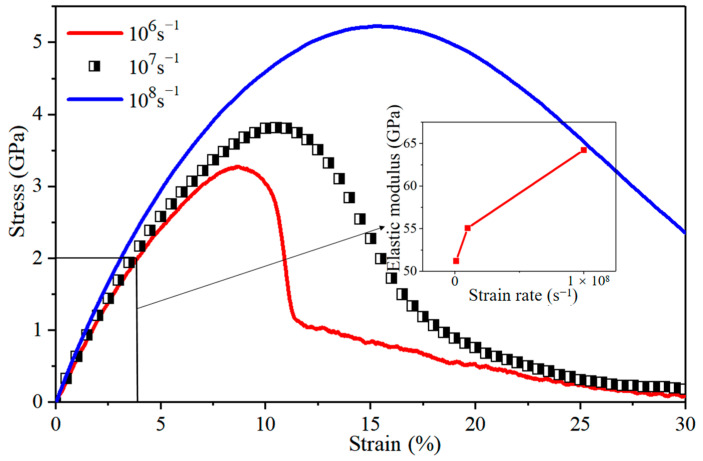
Stress–strain curves for nano-polycrystalline Al/Mg layered composites without IMCs interlayers at different strain rates of 10^6^ s^−1^, 10^7^ s^−1^, and 10^8^ s^−1^, respectively.

Knowledge of whether the deformation characteristics of polycrystalline Mg and Al change is required to determine when they are combined, since they do so at different strain rates. The stress–strain curves of Al/Mg layered composites without IMCs and with the grain size of 6.29 nm at different strain rates of 10^6^ s^−1^, 10^7^ s^−1^, and 10^8^ s^−1^, respectively, are shown in Figure 11. It is noted that the elastic modulus and tensile strength of these situations exhibit an upward trend with the increasing strain rate, and the yield stress also demonstrates an increase with higher strain rates.

**Figure 12:**
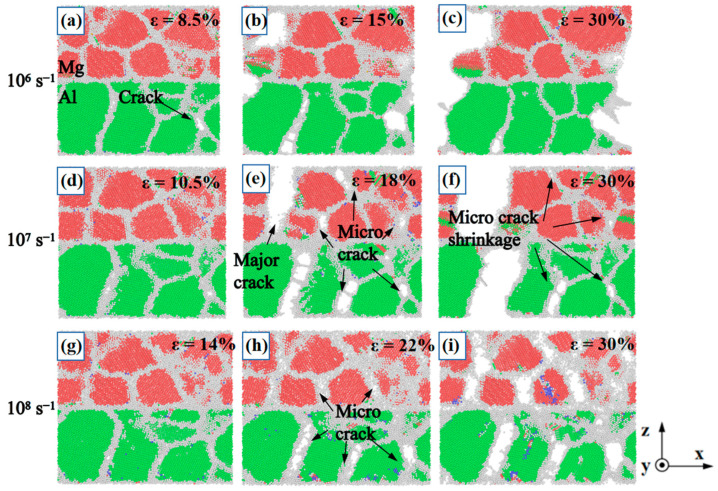
Crack initiation and propagation for nano-polycrystalline Al/Mg layered composites at different strain rates of (**a**–**c**) 10^6^ s^−1^, (**d**–**f**) 10^7^ s^−1^, and (**g**–**i**) 10^8^ s^−1^.

Crack initiation and propagation, observed by intercepting the specific regions where the crack initiation occurs for nano-polycrystalline Al/Mg layered composites at different strain rates of 10^6^ s^−1^, 10^7^ s^−1^ and 10^8^ s^−1^, respectively, are shown in Figure 12. Cracks start to develop at a strain of 8.5%, 10.5%, and 14% for layered composites at strain rates of 10^6^ s^−1^, 10^7^ s^−1^, and 10^8^ s^−1^, respectively. As shown in Figure 12b,c, when subjected to a strain rate of 10^6^ s^−1^, the cracks in Al/Mg layered composite propagate along the initial major cracks until fracturing. At a strain rate of 10^7^ s^−1^, as shown in Figure 12e,f, the major crack continues to expand and eventually fractures, while the micro cracks shrink due to the stress reduction. At a strain rate of 10^8^ s^−1^, as shown in Figure 12h,i, the micro cracks expand along each GB and fracture almost like a fragment, eventually splitting into individual grains. It is demonstrated that strain-rate-driven deformation affects the fracture behaviour of Al/Mg layered composites. The response time of the material to strain is extremely short at very large strain rates and results in more localised strain and ultimately an increase in yield stresses and fracture behaviour at almost every GB that is not parallel to the heterogeneous interface.

**Figure 13:**
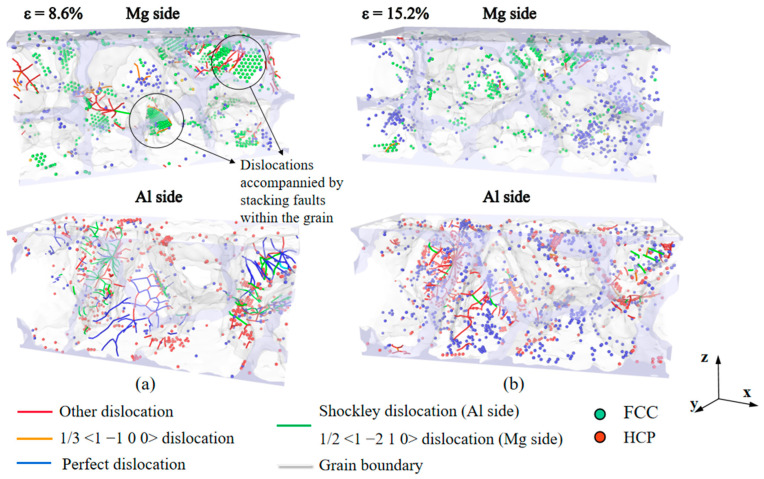
Stacking faults and dislocations in Al/Mg layered composites which reach the yield stress under different strain rates of (**a**) 10^6^ s^−1^ and (**b**) 10^8^ s^−1^.

Figure 13 shows the structural evolution of the Al/Mg layered composites, reaching their yield stresses at strains of 8.6% and 15.2% for strain rates of 10^6^ s^−1^ and 10^8^ s^−1^, respectively. The types of lattices in Al and Mg are different, and in order to observe the changes in dislocations within the grains more intuitively and clearly, the deformations on the Al side and the Mg side were observed separately. The stacking faults appear inside the grains on the Mg side when they are loaded under a strain rate of 10^6^ s^−1^ (Figure 13a), and there are a few 1/3 <1 −1 0 0> dislocations and other dislocations accompanying the stacking faults through the interior of the grains. Additionally, more dislocations also occur at the GBs, namely 1/3 <1 −1 0 0> dislocations and, to a lesser extent, 1/2 <1 −2 1 0> dislocations. In contrast, almost no stacking faults and few dislocations occur within the grains on the Mg side at the strain rate of 10^8^ s^−1^ (Figure 13b), and the dislocations are more evenly distributed at GBs. The dislocations in the composite with the strain rate of 10^6^ s^−1^ are mainly Shockley dislocations, Perfect dislocations, and a few other dislocations, while the dislocations in the composite with the strain rate of 10^8^ s^−1^ are mainly other dislocations, Shockley dislocations, and a few Perfect dislocations. When subjected to a lower strain rate, the dislocation density is significantly higher than when loading at high strain rates. The strain rate affects the deformation mechanism in the Al/Mg layered composites, with the deformation being dominated by stacking faults within grains and the deformation of grains when subjected to a strain rate of 10^6^ s^−1^, while the deformation is dominated by slipping along GBs when loaded at a strain rate of 10^8^ s^−1^.

### 3.4. Combined Effect of Grain Size, Strain Rate, and Interlayer on Deformation Behaviour

**Figure 14:**
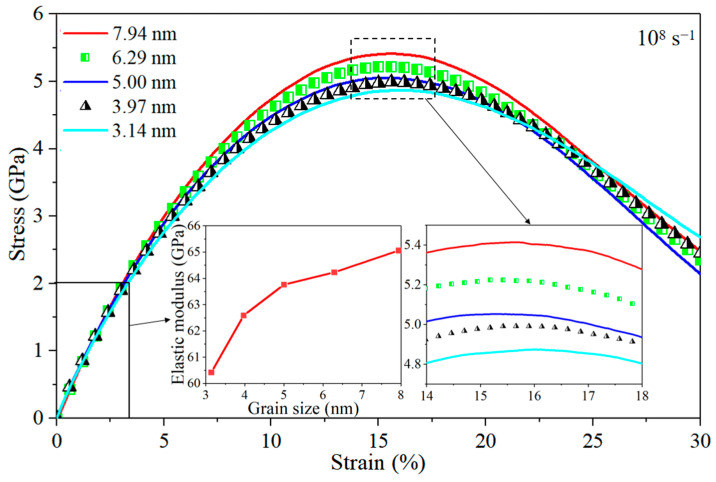
Stress–strain curves for the nano-polycrystalline Al/Mg layered composites with different grain sizes under the strain rate of 10^8^ s^−1^.

The coupling relationship between loading conditions needs to be further studied as the deformation mechanism in nano-polycrystalline Al/Mg layered composites at the atomic scale is not monotonically correlated with external loading conditions including grain size, strain rate, and the interlayer. Stress–strain curves for the Al/Mg layered composites with different grain sizes at a higher strain rate of 10^8^ s^−1^ are shown in Figure 14. Similar to the tendency seen at comparatively lower strain rate of 10^6^ s^−1^, the elastic modulus drops as grain size decreases (Figure 2). The yield stress also exhibits a positive relationship with grain size, with larger grain sizes resulting in higher yield stress, which is contrary to composites subjected to a lower strain rate of 10^6^ s^−1^. In addition, when loading under the strain rate of 10^8^ s^−1^, there is little difference in toughness between the different-grain-size composites, all reaching a yield stress at a strain of around 15%.

**Figure 15:**
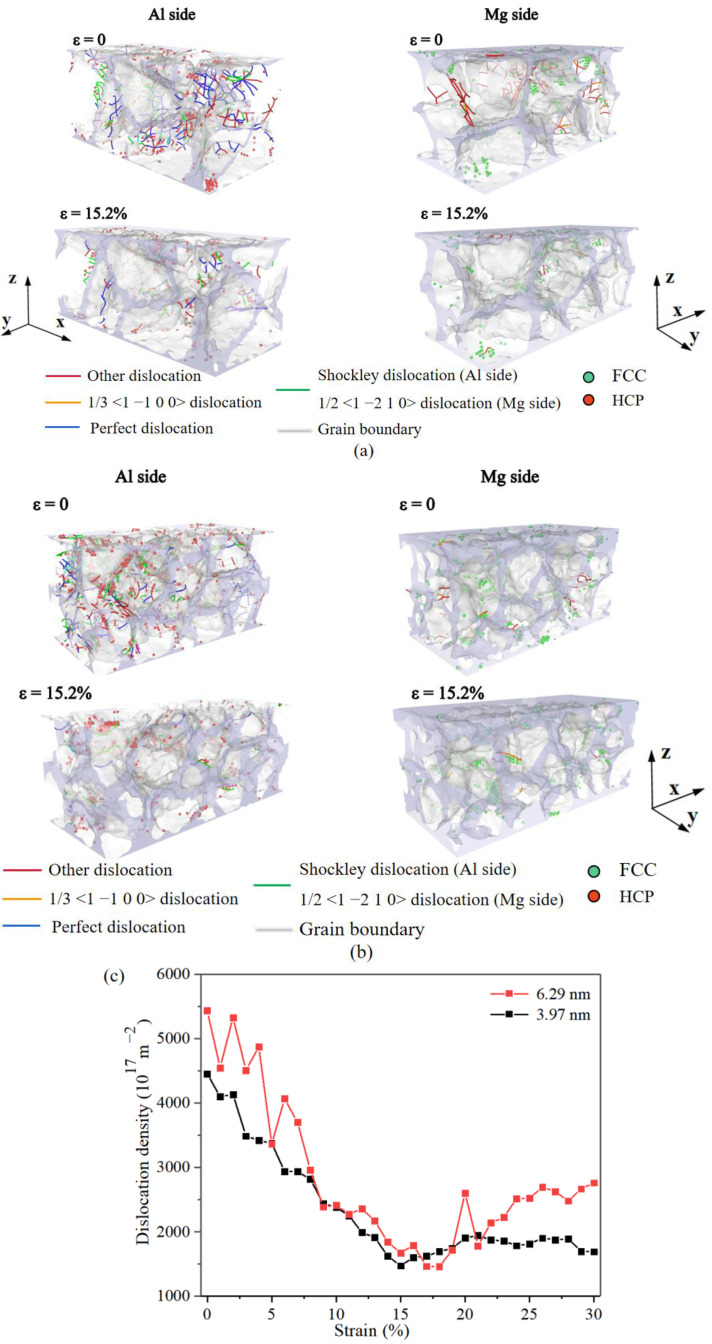
Dislocations in Al/Mg layered composites under the strain rate of 10^8^ s^−1^ with different grain sizes of (**a**) 6.29 nm and (**b**) 3.97 nm, and (**c**) comparison of dislocation density of composites in (**a**,**b**).

As mentioned in the previous section, the deformation mechanism in Al/Mg layered composites changes from predominantly grain deformation to predominantly GB coordinated deformation when the strain rate is changed from 10^6^ s^−1^ to 10^8^ s^−1^ with a constant grain size. A comparison was made between the dislocations within the composites with grain sizes of 6.29 nm and 3.97 nm under loading at a strain rate of 10^8^ s^−1^. A large number of Shockley, Perfect, and other dislocations were generated at the GBs on the Al side and 1/3 <1 −1 0 0> dislocations, and other dislocations on the Mg side (Figure 15a,b), and the dislocation density decreased when the strain reaches 15.2%. As can be seen from Figure 15c, the dislocation density of the composite with a grain size of 6.29 nm is higher than that of the composite with a grain size of 3.97 nm. The dislocation density trend, when compared to Figure 14, is opposite to that of stress–strain, demonstrating that high strain rate dislocations at GBs are the primary impediment to deformation. The smaller the grain size, the lower the dislocation density, ultimately leading to a decrease in yield stress with decreasing grain size, which was previously validated by Ruestes et al. [55] in nano-polycrystalline Zr, and corresponds to the model presented by Marygin [56].

**Figure 16:**
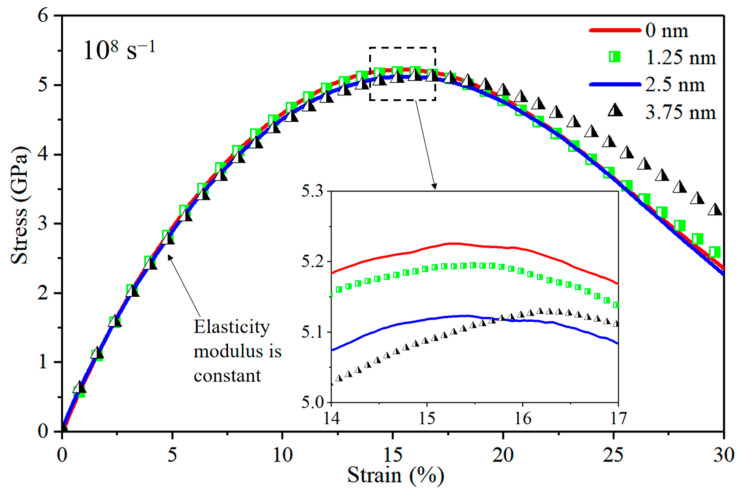
Stress–strain curves for nano-polycrystalline Al/Mg layered composites with different IMCs interlayers thicknesses at a strain rate of 10^8^ s^−1^.

Figure 16 depicts stress–strain curves for Al/Mg layered composites with varying thicknesses of IMCs interlayers, subjected to a strain rate of 10^8^ s^−1^. Notably, the strengthening effect of the IMCs diminishes significantly as the strain rate reaches 10^8^ s^−1^. This phenomenon can be attributed to the challenges faced by grains in accommodating substantial large strains within a limited timeframe under high strain rates. Consequently, the grains struggle to undergo elastic deformation, dislocation, or slip, resulting in rapid cracking at GBs, and this pattern can be supported by the findings in Figure 12i.

## 4. Conclusions

This study investigated the plastic deformation behaviour of nanoscale polycrystalline Al/Mg layered composites under different conditions using MD simulations. Based on the analysis of the observed results, the following main conclusions can be drawn:

1. At the relatively low strain rate of 10^6^ s^−1^, the relationship between grain size and strength in the Al/Mg multi-layered composites adheres to a Hall–Petch pattern. Stacking faults within large grains contribute to stress fluctuations in this scenario. Conversely, at a higher strain rate of 10^8^ s^−1^, the elastic modulus of the composite demonstrates a continuous increase with the growing grain size.
2. The interlayer of IMCs can hinder crack extension and reduce stress concentration, but excessive thickness of the IMCs can compromise the overall strength of the composite. The interlayer with a thickness of 1.25 nm demonstrates optimal enhancement in the tensile strength of the Al/Mg multi-layered composites. It is noteworthy that the reinforcing effect of the IMCs interlayer is weakened when the strain rate rises from 10^6^ s^−1^ to 10^8^ s^−1^.
3. The Al/Mg multi-layered composites exhibit an increase in both the elastic modulus and yield stress as the strain rate elevates. At a strain rate of 10^6^ s^−1^, internal grain deformation prevails as the dominant deformation mechanisms, while at 10^8^ s^−1^, sliding along the GBs becomes the primary mechanism within the composite.

## Data Availability

The data presented in this study are available on request from the corresponding author.

## References

[1] Gu D., Shi X., Poprawe R., Bourell D.L., Setchi R., Zhu J. (2021). Material-structure-performance integrated laser-metal additive manufacturing. Science.

[2] Trost C.O., Žák S., Ruderes K., Hammer R., Rosc J., Krivec T., Schell N., Gänser H.P., Hohenwarter A., Cordill M.J. (2023). Fatigue life assessment of metal foils in multifunctional composites via combined experiments and simulations. Compos. Part B Eng..

[3] Li Z., Cao L., Huo M., Jiang Z. (2023). Evidence-based uncertainty quantification for bending properties of bimetal composites. Appl. Math. Model..

[4] Samal P., Vundavilli P.R., Meher A., Mahapatra M.M. (2020). Recent progress in aluminum metal matrix composites: A review on processing, mechanical and wear properties. J. Manuf. Process..

[5] Zhang C., Lu C., Pei L., Li J., Wang R. (2021). The wrinkling and buckling of graphene induced by nanotwinned copper matrix: A molecular dynamics study. Nano Mater. Sci..

[6] Li Z., Mo H., Tian J., Li J., Hu X., Xia S., Lu Y., Jiang Z. (2024). A novel Ti/Al interpenetrating phase composite with enhanced mechanical properties. Mater. Lett..

[7] Gao T., Wang X., Liu C., Xiao J., Zhao C. (2023). Experimental study on the influence of lamination parameters on the vibration characteristics of variable stiffness composite laminates. Compos. Struct..

[8] Liu T., Song B., Huang G., Jiang X., Guo S., Zheng K., Pan F. (2022). Preparation, structure and properties of Mg/Al laminated metal composites fabricated by roll-bonding, a review. J. Magnes. Alloys.

[9] Lin F., Ren M., Wu H., Lu Y., Yang M., Chen Z., Jiang Z. (2023). Characterisation of microstructure, microhardness and tribological properties of Al matrix hybrid nanocomposites reinforced with B4C and in-situ GNSs. Wear.

[10] Wang C., Ding K., Song Y., Jia X., Lin N., Duan J.a. (2024). Femtosecond laser patterned superhydrophobic surface with anisotropic sliding for droplet manipulation. Opt. Laser Technol..

[11] Kunčická L., Kocich R. (2022). Optimizing electric conductivity of innovative Al-Cu laminated composites via thermomechanical treatment. Mater. Des..

[12] Xing B.-h., Huang T., Song K.-x., Xu L.-j., Xiang N., Chen X.-w., Chen F.-x. (2022). Effect of electric current on formability and microstructure evolution of Cu/Al laminated composite. J. Mater. Res. Technol..

[13] Lu W., Zhang Y., Guo Z., Liu Y., Yu D. (2023). Double laser welding of TC4 alloy and 304 stainless steel using an explosion welded composite plate as an interlayer. Mater. Lett..

[14] Mo T., Xiao H., Lin B., Li W., Wang P., Ma K. (2022). Improvement of mechanical properties of tri-metallic 7075Al/1060Al/304 SS composite via collaborative strengthening behavior. Mater. Sci. Eng. A.

[15] Bak J.H., Kim Y.D., Hong S.S., Lee B.Y., Lee S.R., Jang J.H., Kim M., Char K., Hong S., Park Y.D. (2008). High-frequency micromechanical resonators from aluminium–carbon nanotube nanolaminates. Nat. Mater..

[16] Lin F., Ren M., Jia F., Huo M., Yang M., Chen Z., Jiang Z. (2023). Achieving balanced strength-ductility of heterostructured TiC/graphene nanoplatelets (GNPs) reinforced Al matrix composites by tuning TiC-to-GNPs ratio. Compos. Commun..

[17] Cao Y., Ni S., Liao X., Song M., Zhu Y. (2018). Structural evolutions of metallic materials processed by severe plastic deformation. Mater. Sci. Eng. R Rep..

[18] Zeng L.F., Gao R., Fang Q.F., Wang X.P., Xie Z.M., Miao S., Hao T., Zhang T. (2016). High strength and thermal stability of bulk Cu/Ta nanolamellar multilayers fabricated by cross accumulative roll bonding. Acta Mater..

[19] Kumar D., Phanden R.K., Thakur L. (2021). A review on environment friendly and lightweight Magnesium-Based metal matrix composites and alloys. Mater. Today Proc..

[20] Wang J., Guan F., Jiang W., Li G., Zhang Z., Fan Z. (2021). The role of vibration time in interfacial microstructure and mechanical properties of Al/Mg bimetallic composites produced by a novel compound casting. J. Mater. Res. Technol..

[21] Zhang W., Xu J. (2022). Advanced lightweight materials for Automobiles: A review. Mater. Des..

[22] Jiang S., Bai L., An Q., Yan Z., Li W., Ming K., Zheng S. (2022). Dependence of Plastic Stability on 3D Interface Layer in Nanolaminated Materials. Acta Metall. Sin. (Engl. Lett.).

[23] Li Z., Lin Y.C., Zhang L., Zheng J., Zhao J., Wang R., Jiang Z. (2022). In-situ investigation on tensile properties of a novel Ti/Al composite sheet. Int. J. Mech. Sci..

[24] Yan Z., Liu Z., Yao B., An Q., Zhang R., Zheng S. (2023). Effect of amorphous complexions on plastic deformation of nanolayered composites. Scr. Mater..

[25] Xiangyu G., Wenquan N., Wenle P., Zhiquan H., Tao W., Lifeng M. (2023). Deformation behavior and bonding properties of Cu/Al laminated composite plate by corrugated cold roll bonding. J. Mater. Res. Technol..

[26] Wang X., Meng B., Han J., Wan M. (2023). Effect of grain size on superplastic deformation behavior of Zn-0.033 Mg alloy. Mater. Sci. Eng. A.

[27] Savaedi Z., Mirzadeh H., Aghdam R.M., Mahmudi R. (2022). Effect of grain size on the mechanical properties and bio-corrosion resistance of pure magnesium. J. Mater. Res. Technol..

[28] Fu Q., Wang C., Wu C., Wu Y., Dai X., Jin W., Guo B., Song M., Li W., Yu Z. (2022). Investigating the combined effects of wide stacking faults and grain size on the mechanical properties and corrosion resistance of high-purity Mg. J. Alloys Compd..

[29] Sun J.L., Trimby P.W., Yan F.K., Liao X.Z., Tao N.R., Wang J.T. (2013). Grain size effect on deformation twinning propensity in ultrafine-grained hexagonal close-packed titanium. Scr. Mater..

[30] Liu X. (2022). Investigation on the Mechanical Properties of Polycrystalline Mg Using Molecular Dynamics Simulation. Comput. Model. Eng. Sci..

[31] Chandiran E., Ogawa Y., Ueji R., Somekawa H. (2023). An inverse Hall-Petch relationship during room-temperature compression of commercially pure magnesium. J. Alloys Compd..

[32] Krywopusk N.M., Kecskes L.J., Weihs T.P. (2021). The effect of strain rate on the microstructural evolution of pure Mg during ECAE. Mater. Charact..

[33] Lin Z., Pang W., Xin K., Feng X., Yin F. (2021). The effect of loading strain rates on deformation behavior of Cu/Fe composite. Phys. Lett. A.

[34] Bian X., Wang A., Xie J., Liu P., Mao Z., Chen Y., Liu Z., Gao Y. (2023). Atomic-scale deformation mechanisms of nano-polycrystalline Cu/Al layered composites: A molecular dynamics simulation. J. Mater. Res. Technol..

[35] Ji C., Cai X., Zhou Z., Dong F., Liu S., Gao B. (2021). Effects of intermetallic compound layer thickness on the mechanical properties of silicon-copper interface. Mater. Des..

[36] Li Z., Zhao J., Jia F., Lu Y., Zhang Q., Jiao S., Jiang Z. (2020). Analysis of flow behaviour and strain partitioning mechanism of bimetal composite under hot tensile conditions. Int. J. Mech. Sci..

[37] Li J., Lu C., Pei L., Zhang C., Wang R., Tieu K. (2019). Atomistic simulations of hydrogen effects on tensile deformation behaviour of [0 0 1] twist grain boundaries in nickel. Comput. Mater. Sci..

[38] Xue C., Li S., Chu Z., Yang Q., Li Y., Ma L., Tuo L. (2022). Molecular dynamics study on the effect of temperature on HCP→FCC phase transition of magnesium alloy. J. Magnes. Alloys.

[39] Yang W., Ayoub G., Salehinia I., Mansoor B., Zbib H. (2017). Multiaxial tension/compression asymmetry of Ti/TiN nano laminates: MD investigation. Acta Mater..

[40] Zhang R.F., Germann T.C., Liu X.Y., Wang J., Beyerlein I.J. (2014). Layer size effect on the shock compression behavior of fcc–bcc nanolaminates. Acta Mater..

[41] Tian Y.-Y., Li J., Hu Z.-Y., Wang Z.-P., Fang Q.-H. (2017). Molecular dynamics study of plastic deformation mechanism in Cu/Ag multilayers. Chin. Phys. B.

[42] Hirel P. (2015). Atomsk: A tool for manipulating and converting atomic data files. Comput. Phys. Commun..

[43] Nouri N., Ziaei-Rad V., Ziaei-Rad S. (2012). An approach for simulating microstructures of polycrystalline materials. Comput. Mech..

[44] Plimpton S. (1995). Fast Parallel Algorithms for Short-Range Molecular Dynamics. J. Comput. Phys..

[45] Stukowski A. (2010). Visualization and analysis of atomistic simulation data with OVITO–the Open Visualization Tool. Model. Simul. Mater. Sci. Eng..

[46] Stukowski A., Bulatov V.V., Arsenlis A. (2012). Automated identification and indexing of dislocations in crystal interfaces. Model. Simul. Mater. Sci. Eng..

[47] Shao W., Wu S., Yang W., He J., Lu S., Xu D., Chen J. (2023). Effect of modulation period on microstructure and mechanical properties of (AlSiTiVNbCr)N/(AlSiTiVNbCr)CN nano-multilayer films. Vacuum.

[48] Lu Y.Y., Kotoka R., Ligda J.P., Cao B.B., Yarmolenko S.N., Schuster B.E., Wei Q. (2014). The microstructure and mechanical behavior of Mg/Ti multilayers as a function of individual layer thickness. Acta Mater..

[49] Naoki M., Takanori H., Susumu O. (2022). Molecular dynamics study of interactions between prismatic <a> slip and oxygen-segregated twin boundaries in α-Ti. Materialia.

[50] Ackland G.J. (1992). Theoretical study of titanium surfaces and defects with a new many-body potential. Philos. Mag. A.

[51] Srinivasan P., Nicola L., Simone A. (2017). Modeling pseudo-elasticity in NiTi: Why the MEAM potential outperforms the EAM-FS potential. Comput. Mater. Sci..

[52] Kim Y.-M., Kim N.J., Lee B.-J. (2009). Atomistic Modeling of pure Mg and Mg–Al systems. Calphad.

[53] Jang H.-S., Lee J.-K., Tapia A.J.S.F., Kim N.J., Lee B.-J. (2022). Activation of non-basal <c + a> slip in multicomponent Mg alloys. J. Magnes. Alloys.

[54] Lei J., Ma L., Cai Z., Jia W., Zhi C., Yuan Y., Pan H., Xie H. (2023). Interfacial fracture characteristics of Mg/Al composite plates with different thickness ratios by asymmetrical rolling with differential temperature rolls. Mater. Sci. Eng. A.

[55] Ruestes C., Bertolino G., Ruda M., Farkas D., Bringa E. (2014). Grain size effects in the deformation of [0 0 0 1] textured nanocrystalline Zr. Scr. Mater..

[56] Masumura R.A. (1998). Yield stress of fine grained materials. Acta Mater..

